# The Physician as Naturalist

**Published:** 1889-06-29

**Authors:** 


### The Physician as Naturalist.

The memoirs* contained in this volume form a portion of a number of contributions to professional literature scattered in various journals and now collected under one cover. The first paper is headed "The Physician as Naturalist." The physician, according to Hippocrates, should be the servant of Nature. But it has been said that Hippocrates, as the servant of Nature, was simply a "waiter upon death"; for Nature is too often bent, not on healing a man, but on putting him into his coffin. And so the successors of Hippocrates, the physicians of the middle ages, resorted to astronomy, "magike naturel," the black art, and simples, whereby to control Nature, without any attempt at genuine research into the nature of diseases as we now understand it in modern physic. There is still, however, something wanting in the training of the physician, especially in regard to early education in physics and natural science. While the physician must ever be a student of Nature, he must not be servile to Nature. There are times and seasons when he may leave much to Nature, but there are also times and seasons when he should not hesitate to interfere.

Dr. Gairdner's second paper is headed "Has the Art of Medicine Advanced during the Last Hundred Years?" Of course, this question must be answered in the affirmative. The author shows that in the period in question we have got rid of Brunoniaism, which classified disease as sthenic and asthemic. We have also discarded blood-letting. No physician now follows the old Hamiltonian system of prescribing the pill and black draught for every disease. And we have seen the rise and fall of routine stimulation as advocated by Todd. Lastly, mercury is used in the treatment of disease with extreme caution, instead of being "exhibited" recklessly to salivation as in former times. On the other hand, we now have confidence in the modern use of such agents as chloroform, bromide of potassium, digitalis, strophanthus, and the salicyl compounds, etc., while researches into the origin and nature of various diseases has tended to direct the use of modern therapeutical agents in the path of stability. Then we employ hygienic remedies not used in former years, the progress of preventive medicine, otherwise called sanitary science, during the last thirty years having steadily advanced on the same lines of improvement as curative medicine. In article No. VIII., "On Sanitary Science and Preventive Medicine," the author pays a tribute to the genius of Dr. Farr, "who at last solved the problem of investing numbers with a correspondingly real interest, and made blue books among the most stimulating and suggestive productions of the age." "The present aspect of medical science and old orthodoxy, and modern opinion," is the title of a further paper, which treats on much the same subjects as the last. The author announces that the day of orthodoxies is over, and the day of real science has dawned. Still, however, the study of medicine remains to a large extent governed by precedents derived from the past, and the author is not on the side of those who, because they have cast aside the fetters of the middle ages, would forget that medicine has a history. Certainly, as regards closeness and accuracy in the description of the symptoms of disease, we are not much in advance of olden authors.

A book of the kind would scarcely be complete unless it contained an essay on homœopathy. In an article on that subject, Dr. Gairdner first gives the laws of homœopathy, as propounded by Hahnemann. The fundamental doctrine of homœopathy was that diseases are to be cured by medicines which are capable of exciting closely similar symptoms in healthy persons, and that such medicines are to be given in infinitesimal doses. These homœopathic laws are, according to Hahnemann, of universal application in theory, and are limited in practice only by the limited number of remedies, and by our imperfect knowledge of their action upon the human body. But Hahnemann also held that "psoric miasm," of which itch is the outward and visible form, was at the root of all chronic diseases. Dr. Gairdner examines all Hahnemann's assertions at some length, submitting them to a critical analysis, so that not even a metaphorical morsel of bread remains in the metaphorical bushel of sack. For example, Hahnemann did not know that itch is caused by an insect, for the "acarus scabeii" had not then been discovered. Again, with regard to homœopathic dilutions, Dr. Gairdner shows that at the thirtieth dilution—the ordinary one employed by Hahnemann—a grain of medicine would be dissolved in a quantity of liquid "equal to many hundred spheres, each with a radius extending from the earth to the nearest fixed star." Dr. Gairdner might also have added that what is true of one stimulant must be correct as regards another; therefore, a drop of brandy diluted as above ought to be more powerful than a whole bottleful!

Dr. Gairdner need not, however, have troubled himself to demolish a glass bead with a steam hammer. Modern disciples of homœopathy have so fallen away from the original doctrines, that they have little claim to be termed homœopaths. For example, a recent writer while still asserting the doctrine of *similia similibus curentur*, says, regard must be had to the seat and pathology of the disease, and that the drug should be used which will affect the organ in fault. He therefore adds, the "science" of "Organopathy" to homœopathy. Hahnemann maintained that cures were never effected excepting by medicines having power to produce similar complaints. But the "law" *similia similibus curentur* has been compromised for that of contrariety, some so-called homœopaths having asserted an exactly opposite formula—viz., *contraria contrariis curentur.* Moreover, so-called homœopaths do not disdain to employ allopathic medicines, using purgatives, sudorifics, diuretics, etc., to such an extent that an homœopathic journal not very long since plaintively wailed this backsliding. Indeed, Dr. Wyld, writing so far back as 1877, stated infinitesimal doses were practically abandoned.

It was an essential part of Hahnemann's teaching that Nature is a bad physician, and not to be trusted, but practically homœopathy implies a complete trust in Nature, for whatever may be asserted to the contrary, it has been sufficiently proved by experiment that the diluted medicines of homœopathy have no effect whatever on the human constitution. But as many diseases tend to terminate in health, homœopathy has been credited with the cure, which really was *post* and not *propter* the homœopathic "remedies." Lastly, it may be observed that although Hahnemann is generally regarded as the founder of homœopathy he was anticipated by Hippocrates, who said some diseases were best treated by similars. There is, indeed, nothing new under the sun.

We have not space for more than the mention of other articles comprised in this series of essays. Such are "Facts and Conclusions as to the use of Alcohol in Typhus Fever"; "Course of Typhus Fever, and Phenomena of the Crisis"; "Two Months of Fever Duty in the Glasgow Infirmary"; "Mind and Body"; "The Progress of Pathological Science," and a tribute to the memory of the late Dr. Alison.

Before closing our notice of Dr. Gairdner's volume, we venture to remark that we should scarcely have thought it worth while for a man of Dr. Gairdner's eminence to republish these papers in the form of a book. As more than one relates to the same or to similar subjects, considerable repetition is involved. And as a matter of fact, there is very little in the volume which is not familiar to every medical man, or at least should be so known. Nevertheless, should anyone desire information with regard to the subjects treated, a better series of articles could not be found.

* "Addresses and Memoirs bearing on the History and Progress of Medicine, chiefly during the last Hundred Years." By W. T. Gairdner, M.D., LL.D., Professor of Medicine in the University of Glasgow; President of the British Medical Association; Physician in Ordinary to H.M. the Queen in Scotland. James Maclehose and Sons, Glasgow 1889

